# One in the Dance: Musical Correlates of Group Synchrony in a Real-World Club Environment

**DOI:** 10.1371/journal.pone.0164783

**Published:** 2016-10-20

**Authors:** Melissa Ellamil, Joshua Berson, Jen Wong, Louis Buckley, Daniel S. Margulies

**Affiliations:** 1 Max Planck Research Group for Neuroanatomy and Connectivity, Max Planck Institute for Human Cognitive and Brain Sciences, Leipzig, Germany; 2 Guerilla Science, London, United Kingdom; University of Pittsburgh, UNITED STATES

## Abstract

Previous research on interpersonal synchrony has mainly investigated small groups in isolated laboratory settings, which may not fully reflect the complex and dynamic interactions of real-life social situations. The present study expands on this by examining group synchrony across a large number of individuals in a naturalistic environment. Smartphone acceleration measures were recorded from participants during a music set in a dance club and assessed to identify how group movement synchrony covaried with various features of the music. In an evaluation of different preprocessing and analysis methods, giving more weight to front-back movement provided the most sensitive and reliable measure of group synchrony. During the club music set, group synchrony of torso movement was most strongly associated with pulsations that approximate walking rhythm (100–150 beats per minute). Songs with higher real-world play counts were also correlated with greater group synchrony. Group synchrony thus appears to be constrained by familiarity of the movement (walking action and rhythm) and of the music (song popularity). These findings from a real-world, large-scale social and musical setting can guide the development of methods for capturing and examining collective experiences in the laboratory and for effectively linking them to synchrony across people in daily life.

## Introduction

Dancing with others to music is a universal social activity that is central to many cultural and religious rituals as well as various forms of creative expression. Coordinated rhythmic movement is theorized to have evolved to establish and maintain group cohesion, promoting enhanced coordination during survival-relevant activities such as hunting or warfare [1–3]. Psychological experiments have shown that synchrony of movement among individuals appears to blur the boundary between self and other, fostering social rapport and increasing group cooperation [4–6]. Similarly, anthropological field studies have observed that physical synchrony during large-scale social gatherings, such as Andaman Islanders performing dance rituals [7], street revelers during Carnival [8], and ravers dancing to beat-heavy music [9], lead to feelings of being one with the community or “collective effervescence” [10]. However, although the effects of physical coordination across a group of individuals have been extensively studied, the features of the environment that affect group synchrony of movement have received considerably less attention.

Most prior studies of interpersonal synchrony have investigated coordination between only two people, while a handful of more recent experiments have assessed small groups of five to seventeen people. These investigations of group synchrony show that shared social experiences are reflected in coordinated physiological responses. For example, active participants and related spectators, but not unrelated audience members, displayed synchronized arousal, as measured by heart rate, during a Spanish fire-walking ritual [11]. Choir members also demonstrated higher heart rate and breathing synchrony during singing relative to resting and when singing together compared to singing different voice parts [12]. Similarly, participants assessed collectively in a group, compared to those examined individually, showed greater synchronization of breathing along with spontaneous coordination of arm swinging [13]. In addition, participants demonstrated synchronized brain activation patterns in various sensory and association areas in response to certain features of naturalistic stimuli, such as emotionally arousing scenes in a movie [14], extended excerpts of symphonic music compared to pseudomusical sequences [15], and multisensory versus unisensory extended dance recordings [16]. However, with the exception of one field study with 38 participants [11], these experiments have mainly investigated small groups in isolated laboratory settings, which may not fully reflect the complex and dynamic interactions of real-life social situations. None have assessed group synchrony of movement during dance.

The present study expands on previous research by examining the influence of various musical features on movement synchrony across a large number of individuals in a naturalistic dance environment. Movement data from 46 club-goers were recorded during a music set at a dance event using an accelerometer app installed on mobile phones placed in running belts. Different methods of processing movement data and quantifying group synchrony were then assessed for discriminability and reliability using similarly acquired acceleration measures from a test session that alternated between conditions of high (i.e., attention to music and similar dance sequences) and low (i.e., ignoring the music and independent dance sequences) synchrony. The most sensitive and reliable preprocessing and analysis combination was then used to evaluate group synchrony of movement during the dance event and to examine how it covaried with different features of the music played, including popularity, rhythm, timbre, and pitch. Group synchrony appeared to be constrained by familiarity of the movement and of the music, with greater synchrony of torso movement associated with music that approximated walking rhythm and popular songs as measured by real-world play counts.

## Materials and Methods

### Dance club data acquisition

#### Participants

Out of the 75 individuals (47 female and 28 male; *M* = 31.75 years old, *SD* = 6.90, *range* = 20–49) who participated in the experiment, 46 participants (33 female and 13 male; *M* = 31.63 years old, *SD* = 7.12, *range* = 20–49) had recorded movement data for the entire duration of the experiment and were included in the analyses. Participants were recruited through an online registration form, during which they gave informed consent to participate and answered a demographics questionnaire. They received free entry to the dance club as compensation as well as free dance warm-up activities and disco accessories at the event. All protocols were approved by the Ethics Advisory Sub-Committee in the Department of Psychology at Durham University (Durham, UK).

#### Materials

Movement data for each participant were collected using an accelerometer app installed on a mobile phone placed in one pocket of a running belt. The Physics Toolbox Accelerometer app (v1.3.7; Vieyra Software) measured and stored acceleration force excluding the force of gravity (i.e., linear acceleration) applied to the mobile phone on the *x*, *y*, and *z* physical axes in m/s^2^ units. The HTC One M7 mobile phones used (Android OS v4.1.2 –v5.0.2; 2GB RAM and 1.7GHz CPU; HTC Corporation) were held on the front and center of the waist in Friendly Swede Dual-Pocket Running Belts (waist fits 27”– 43”; The Friendly Swede). With the *Fastest* sampling rate option chosen in the accelerometer app, the devices sampled linear acceleration data at an average rate of 54.18 Hz (*SD* = 4.13, *range* = 47.67–63.49).

#### Procedures

The experiment was conducted during a dance event called *Science of Disco* held on the evening of December 5, 2015 by Carwash, London’s longest running disco club (LOOP, 19 Dering Street, London, UK). Data collection started at 8:33 PM and ended at 9:04 PM, during which the DJ played a sequence of well-known, disco-style tracks. During the hour prior to data collection, after confirming their registration details and informed consent, each participant was fitted with a running belt containing a mobile phone that recorded movement data. The running belt was placed at waist level with the pocket holding the mobile phone centered on the front of the torso, in order to better capture whole-body movement related to interacting with the music, compared to, for example, hand gestures [17] that also accompany speech during conversation outside of musical contexts. The participant was then instructed to act as they normally would in a club on a night out. Through the installed accelerometer app, the mobile phone logged linear acceleration in its *x* (up–down movement, when placed horizontally), *y* (left–right movement, when placed horizontally), and *z* (front–back movement) axes, which was later exported in a comma-separated value file with millisecond-scale timestamps based on the device’s clock synchronized to network time.

### Discriminability and reliability test data

#### Participants

Six volunteers (4 female and 2 male; *M* = 27.00 years old, *SD* = 3.63, *range* = 23–32) gave informed consent and participated in the discriminability and reliability testing of preprocessing and analysis pipelines for measuring group movement synchrony. The volunteers were recruited from the Neuroanatomy and Connectivity Research Group at the Max Planck Institute for Human Cognitive and Brain Sciences (Leipzig, Germany).

#### Materials

Movement data for each participant were collected using an accelerometer app installed on two mobile phones placed in the pockets of a running belt. The Physics Toolbox Accelerometer app (v1.3.7) was set up on 11 HTC Desire C devices (Android OS v4.0.3; 512MB RAM and 600MHz CPU) and 1 Acer Liquid Z410 device (Android OS v4.4.4; 2GB RAM and 1.3GHz CPU; Acer Inc.), as the last HTC Desire C device was not operational. One phone each was placed in the left and right pockets of the Friendly Swede Dual-Pocket Running Belts, the same model used during the *Science of Disco* data collection. With the *Fastest* sampling rate option chosen in the accelerometer app, the devices sampled linear acceleration data at an average rate of 65.09 Hz (*SD* = 4.21, *range* = 63.27–77.76).

#### Procedures

The discriminability and reliability testing of the group movement synchrony preprocessing and analysis pipelines was conducted in an experiment room at the Max Planck Institute for Human Cognitive and Brain Sciences. Half an hour prior to data collection, each participant was fitted with a running belt containing two mobile phones that recorded movement data. The running belt was placed at waist level with the middle space between the two pockets centered on the front of the torso. The participants were then taught a short sequence of beginner disco-style dance steps to be repeated during certain songs. The music used for the session was taken from the *Science of Disco* data acquisition DJ set. The songs were rearranged to match the count of the taught dance sequence and *You Should Be Dancing* by the Bee Gees was added to create an even number of songs.

The experiment alternated between conditions (i.e., songs) of high synchrony, during which participants were instructed to attend to the music and other people while performing similar dance sequences, and low synchrony, during which participants were instructed to ignore the music and other people while performing independent dance sequences. There were five variations of high synchrony blocks (i.e., songs): *choreographed* or same sequence exactly, *anti-phase* or same sequence in opposite directions (i.e., half of the participants mirrored the other half), *different magnitude* or same sequence with different ranges (i.e., half of the participants made smaller movements), *different delay* or same sequence with different lag (i.e., half of the participants moved one count behind the other half), and *non-choreographed* or highly “into” the music but without choreography.

### Preprocessing pipelines

#### Axes combination

Since the signal measured by an individual accelerometer axis reflects only one aspect of movement and depends on the specific placement or orientation of the recording device, the time courses for the three acceleration axes were combined to obtain a global measure of movement over time for each participant. Thus, for each time point, the standard force vector magnitude $\sqrt{x^{2}+y^{2}+z^{2}}$ was calculated, along with force vector magnitudes aligned [18] to the *x*-axis, $arcsin(x\;/\;\sqrt{x^{2}+y^{2}+z^{2}})$; *y*-axis, $arcsin(y\;/\;\sqrt{x^{2}+y^{2}+z^{2}})$; and *z*-axis, $arcsin(z\;/\;\sqrt{x^{2}+y^{2}+z^{2}})$.

#### Time interpolation

The Android API captures accelerometer information based on the displacement of the mobile phone relative to its previous position such that there is sparser sampling during periods of lower variation and vice versa. In addition, because the sampling capability of the accelerometer varies over time and across devices, the Android API only allows four suggested sampling rates, from fastest to slowest: *Fastest* (as fast as possible), *Game* (suitable for games), *Normal* (suitable for screen orientation changes), and *UI* (suitable for the user interface). Thus, movement data for each participant were collected at different time points and different time intervals. To align the participants’ movement data to each other, for a given combined acceleration time course, each data sample was matched to the corresponding millisecond time bin based on the clock time of its recording and was used to interpolate values over the missing time bins. Standard linear, piecewise cubic, and nearest neighbor interpolation methods were assessed. Cubic spline interpolation introduced spurious spikes and dips in the acceleration time courses and was thus not included in the analyses.

#### Data downsampling

Each combined and interpolated acceleration time course was then downsampled, which reduced its interpolated sampling rate back to its actual average sampling rate over time and across devices (i.e., 54.18 Hz for the *Science of Disco* data collection and 65.09 Hz for the discriminability and reliability test session). After linear detrending (to prevent edge artifacts due to filtering before resampling the data) of the combined, interpolated time courses, the averaging and decimation methods for downsampling were performed. The averaging method, which preserves the temporal shape of the waveform, replaces each set of a given number of samples with their mean (e.g., for the discriminability and reliability test session, averaging over 16 millisecond time bins to reduce the interpolated sampling rate to 62.50 Hz, as a conservative approximation of the actual average sampling rate of 65.09 Hz). The decimation method, which preserves the spectral properties of the waveform, first applies a low-pass filter to prevent aliasing of the signal and then discards samples within a given interval (e.g., for the discriminability and reliability test session, after applying a Chebyshev Type I IIR filter of order 8, keeping only every 16th millisecond time bin).

#### Wavelet decomposition

After the downsampled, interpolated, combined time courses were standardized (to center the data and bring them to a common scale), they were decomposed into different frequency sub-bands using maximal overlap discrete wavelet transform (MODWT) to allow evaluation of group synchrony of movement at various time scales. The MODWT (also known as non-decimated DWT, stationary DWT, translation-invariant DWT, and time-invariant DWT) [19] retains downsampled values at each decomposition level that would be discarded with the standard DWT, preserving the number of observations in the actual signal at all frequency sub-bands. Thus, the MODWT is shift invariant, meaning that a small delay between two time series is transformed into a similarly small delay in the decomposed signals, which might be exaggerated with the standard DWT. In addition, unlike the standard DWT which requires time series lengths to be a power of 2 for a complete decomposition, the MODWT can handle arbitrary time series lengths.

Complete MODWT decompositions into all possible frequency sub-bands (i.e., 17 levels for the discriminability and reliability test session and 16 levels for the *Science of Disco* data collection) using Daubechies (*db2*, *db4*, *db6*), Symlet (*sym2*, *sym4*, *sym6*), and Coiflet (*coif1*, *coif3*, *coif5*) mother wavelets were examined. The wavelet name specifies the number of vanishing moments *V* associated with the function, which is related to the number of filter coefficients *K* it uses by *K* = 2*V*. Shorter filter lengths result in better time localization but poorer frequency approximation, while longer filter lengths result in better frequency approximation but poorer time localization (i.e., less accurate detection of rapid signal changes).

### Group synchrony measures

Computation of instantaneous phase synchronization enables the measurement of similarity over time between two signals without the need for a sliding window containing several time points (such as with correlation coefficients), which decreases the temporal resolution of the analysis [20,21]. Instantaneous phase synchronization between two signals is obtained by extracting each signal’s phase time series (separating it from its amplitude time series) and then calculating their phase difference at each time point. The intersubject phase synchronization and cluster phase method indices of group synchrony over time were evaluated. In particular, the present study was primarily concerned with relative differences in group synchrony across song segments and the relationship between group synchrony and music features, rather than its absolute value or significance.

#### Intersubject phase synchronization

To assess time-varying synchrony among three or more signals or participants, the intersubject phase synchronization measure [20,21] averages across the phase difference time series from each pair of participants. For each frequency sub-band of the decomposed, downsampled, interpolated, combined movement time courses from each participant, the phase information time series was extracted after converting the real signal into its corresponding complex analytic signal using the Hilbert transform. The absolute angular distance or phase difference at each time point between the phase time series of each pair of participants was calculated as an indicator of their (dis)similarity over time. The phase difference time courses were then averaged and normalized across all pairs of participants. At each time point, a value of 1 indicated complete phase similarity and a value of 0 indicated lack of phase similarity across participants.

#### Cluster phase method

An alternative way of assessing time-varying synchrony among three or more signals or participants, the cluster phase method [22,23], is based on the individual participant’s phase difference time series relative to the average group phase time series, instead of the phase difference time series between two participants (as with intersubject phase synchronization). As previously described, for each frequency sub-band of the movement time courses from each participant, the phase information time series (*individual phase*) was extracted from its corresponding complex analytic signal computed using the Hilbert transform. The average phase time series across all participants was computed (*cluster phase*) and subtracted from each individual phase time series (*relative phase*). For each participant, the average relative phase across all time points was then calculated (*mean relative phase*) and subtracted from each time point of the relative phase time series. The absolute average of the resulting time series from each participant represented the degree of movement synchrony across the whole group over time. At each time point, a value closer to 1 indicated higher group synchrony, while a value closer to 0 indicated lower group synchrony.

### Discriminability and reliability assessment

To assess the discriminability of a given preprocessing pipeline and synchrony measure combination, the group movement synchrony time course from the test session was correlated with the synchrony condition boxcar time course. The group movement synchrony time course was the average synchrony measure time course across the middle five frequency bands (0.06 Hz– 0.98 Hz for the test session) and was computed on data from the mobile phones in the left running belt pockets of all six volunteers. The synchrony condition boxcar time course was constructed by assigning 1’s to high synchrony time points and 0’s to low synchrony time points. For each group movement synchrony time course, the Pearson correlation coefficient indicated its similarity to the synchrony condition boxcar time course and thus its ability to discriminate between the high and low synchrony conditions during the test session. The preprocessing and analysis combinations with positive (i.e., higher values during synchrony conditions) and significant (i.e., *p* < .05 / 432 combinations) correlations were then sorted in descending order.

To evaluate the reliability of a particular preprocessing pipeline and synchrony measure combination, the group movement synchrony time course computed on data from the mobile phones in the left running belt pockets of five volunteers were compared to the time course computed from phones in the right pockets of the same volunteers. One volunteer was excluded from the analysis because the phone in their right pocked failed to collect accelerometer data. Each of the two group movement synchrony time courses was the average of the corresponding synchrony measure time courses across the middle five frequency bands (0.06 Hz– 0.98 Hz for the test session). For each pair of group movement synchrony time courses, the intraclass correlation coefficient (ICC[3,1] or two-way mixed effects on a single measure) [24] indicated their level of absolute agreement and thus their reliability in measuring the degree of synchrony during the test session. The preprocessing and analysis combinations with sufficient (i.e., *ICC* > .70) and significant (i.e., *p* < .05 / 432 combinations) reliability were then sorted in descending order.

### Music popularity index

To examine the relationship between group movement synchrony and popularity of the music, the total number of plays or “scrobbles” for each song and artist in the *Science of Disco* music set were retrieved from Last.fm (March 14, 2016, 11:30 AM CET). Last.fm is an online database and recommendation service that tracks users’ listening behavior on music streaming websites (e.g., Spotify) and music player applications (e.g., iTunes). Compared to a song’s or artist’s placement on traditional music charts (e.g., Billboard), scrobble numbers are stable over time and reflect the continued popularity of a song or artist independent of current radio play and album sales.

### Music feature extraction

To investigate the relationship between group movement synchrony and various aspects of the music, time courses of features related to dynamics, rhythm, timbre, pitch, and tone were extracted from the audio recording of the *Science of Disco* music set using MATLAB MIRToolbox v1.6.1 [25]. The extracted time courses included: root mean square energy or loudness [26]; tempo; metrical centroid or speed of pulsations [27]; metrical strength or strength of pulsations [27]; pulse clarity [28]; attack slope or percussiveness; zero-crossings or noisiness; high-frequency spectral flux (6400–12800 Hz, produced by cymbals and hi-hats) [29]; low-frequency spectral flux (50–100 Hz, produced by kick drums and bass guitars) [29]; brightness or high frequency energy [30]; roughness or sensory dissonance [31]; spectral irregularity or variability [32]; pitch [33]; inharmonicity or departure from fundamental frequency multiples; mode (major vs. minor scale); harmonic changes [34]; and novelty or significant musical transitions [35].

## Results

### Discriminability and reliability of synchrony analysis parameters

Synchrony of behavioral or physiological responses over time across more than two people has been quantified using several different methods in previous studies of interpersonal coordination. Thus, the effects of various parameters on the ability of different preprocessing and analysis pipelines to discriminate between high and low synchrony conditions and to replicate synchrony measures across different devices during the test session were assessed to guide the selection of the most sensitive and reliable measure of group synchrony of movement during dancing.

A six-way between-subjects analysis of variance was conducted to compare the discriminability of different preprocessing and analysis pipelines, as measured by the (Fisher’s *z*- transformed) correlation or similarity of the group synchrony time course with the condition boxcar time course (i.e., 1’s for high synchrony and 0’s for low synchrony time points) (S1 Fig, top). There was a main effect of group synchrony measure, *F*(1,420) = 171.62, *p* < .001, with correlations significantly higher for pipelines using intersubject phase synchronization (*M* = 0.20, *SD* = 0.36) than the cluster phase method (*M* = –0.07, *SD* = 0.23). There was also a main effect of axes combination, *F*(3,420) = 162.79, *p* < .001, with follow-up multiple *t*-tests using a Bonferroni correction (*α* = .05 / 6 = .0083) showing that the different combination types all significantly differed from each other, *t*(214) > 2.90, *p* < .005. Correlations were the highest for pipelines using *z*-axis aligned combination (*M* = 0.38, *SD* = 0.15), followed by *x*-axis aligned combination (*M* = 0.12, *SD* = 0.18) and standard combination (*M* = –0.003, *SD* = 0.39), while *y*-axis aligned combination showed negative correlations (*M* = –0.24, *SD* = 0.19). There were no main effects of wavelet type (family set and filter length), interpolation method, and downsampling method.

A six-way between-subjects analysis of variance was also conducted to compare the reliability of different preprocessing and analysis pipelines, as measured by the (Fisher’s *z*- transformed) intraclass correlation or absolute agreement between group synchrony time courses from two sets of devices on the same participants (S1 Fig, bottom). There was a main effect of axes combination, *F*(3,420) = 537.84, *p* < .001, with follow-up multiple *t*-tests using a Bonferroni correction (*α* = .05 / 6 = .0083) showing that the different combination types all significantly differed from each other, *t*(214) > 2.96, *p* < .005. Intraclass correlations were the highest for pipelines using *z*-axis aligned combination (*M* = 0.64, *SD* = 0.15), followed by *x*-axis aligned combination (*M* = 0.61, *SD* = 0.11) and standard combination (*M* = 0.30, *SD* = 0.05), while *y*-axis aligned combination showed the lowest absolute agreement (*M* = –0.12, *SD* = 0.30). There were no main effects of group synchrony measure, wavelet type (family set and filter length), interpolation method, and downsampling method.

The group movement synchrony time course most correlated with the synchrony condition boxcar time course (S1 Table) used *z*-aligned axis combination, linear interpolation, decimation downsampling, *db6* wavelet decomposition, and intersubject phase synchronization, *r*(136327) = 0.538, *p* < .001, which ranked 13th on agreement in the test dataset, *ICC* = 0.737, *F*(136328) = 6.65, *p* < .001. The group movement synchrony time course with the highest agreement across two sets of devices on the same participants (S2 Table) used *z*-aligned axis combination, linear interpolation, averaging downsampling, *coif1* wavelet decomposition, and intersubject phase synchronization, *ICC* = 0.752, *F*(136328) = 7.14, *p* < .001, which ranked 6th on correlation, *r*(136327) = 0.533, *p* < .001. Thus, as the best compromise between reliability and discriminability, the latter preprocessing pipeline and synchrony measure combination was used for the analysis of the club dataset.

### Music features and group synchrony of movement during dance

**Fig 1:**
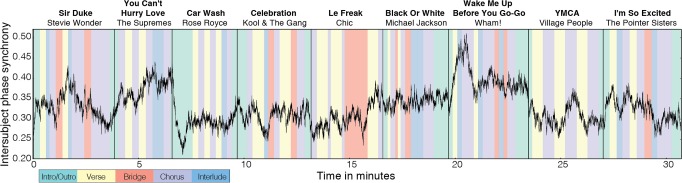
Group synchrony of movement over time during a music set at a dance club. Movement data from participants at the event were preprocessed and analyzed using *z*-aligned axis combination, linear interpolation, averaging downsampling, *coif1* wavelet decomposition, and intersubject phase synchronization. The group movement synchrony time course is divided into the songs played during the music set and segmented into the different parts of a song, including intro and outro, verse, pre-chorus and bridge, chorus, and interlude.

**Fig 2:**
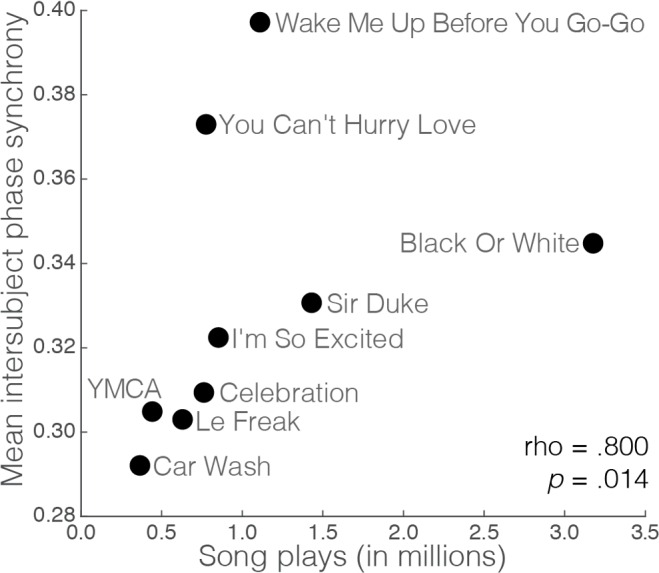
Relationship between group synchrony of movement and song popularity. Play counts from music websites and applications for each song are plotted against their average intersubject phase synchronization values.

Accelerometer data from participants at the dance club event were preprocessed using *z*-aligned axis combination, linear interpolation, averaging downsampling, and *coif1* wavelet decomposition. To obtain a measure of group synchrony of movement over time during the music set, intersubject phase synchronization time courses were then computed for each frequency sub-band of the preprocessed movement time courses and averaged across the middle five frequency bands (0.05 Hz—0.82 Hz for the club session) (Fig 1). Average intersubject phase synchronization was the highest for *Wake Me Up Before You Go-Go* by Wham! (*M* = 0.397, *SD* = 0.036), *You Can’t Hurry Love* by The Supremes (*M* = 0.373, *SD* = 0.028), and *Black Or White* by Michael Jackson (*M* = 0.345, *SD* = 0.015). *YMCA* by the Village People (*M* = 0.305, *SD* = 0.024), *Le Freak* by Chic (*M* = 0.303, *SD* = 0.031), and *Car Wash* by Rose Royce (*M* = 0.292, *SD* = 0.029), meanwhile, had the lowest average intersubject phase synchronization (Table 1). Average intersubject phase synchronization for each song was significantly related (*p* < .05 / 2 = .025) to the song’s popularity (*Spearman’s ρ* = 0.80, *p* = .014), but not to the artist’s popularity (*ρ* = 0.62, *p* = .086), as measured by the number of times the song or artist has been played or “scrobbled” to Last.fm (Fig 2).

**Table 1:** Song synchrony and music popularity.

Song (Artist)	Synchrony (M, SD)	Song Plays	Artist Plays
Wake Me Up Before You Go-Go (Wham!)	0.397 (0.036)	1,108,520	6,533,460
You Can't Hurry Love (The Supremes)	0.373 (0.028)	773,514	6,754,282
Black Or White (Michael Jackson)	0.345 (0.015)	3,174,790	113,888,779
Sir Duke (Stevie Wonder)	0.331 (0.032)	1,427,228	33,124,230
I’m So Excited (The Pointer Sisters)	0.322 (0.028)	850,543	2,855,596
Celebration (Kool & The Gang)	0.309 (0.022)	760,696	7,049,555
YMCA (Village People)	0.305 (0.024)	441,468	1,947,368
Le Freak (Chic)	0.303 (0.031)	628,852	3,267,270
Car Wash (Rose Royce)	0.292 (0.029)	365,335	1,125,535

**Fig 3:**
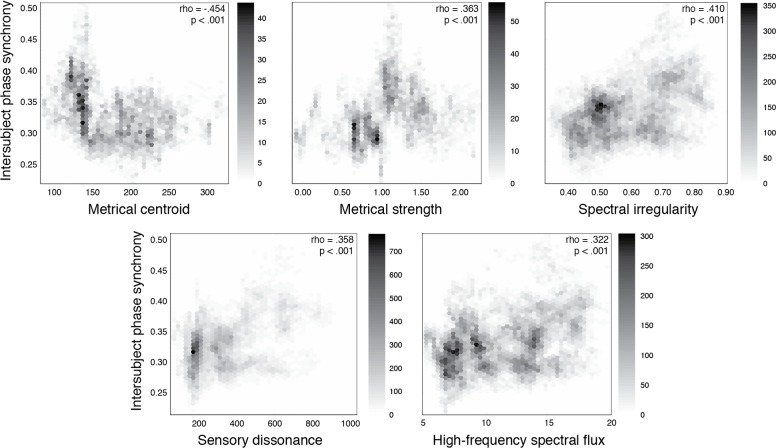
Relationship between group synchrony of movement and different features of the music. Values over the duration of the music set are plotted for intersubject phase synchronization and metrical centroid or speed of pulsations, metrical strength or clarity of pulsations, spectral irregularity or variability, sensory dissonance or roughness, and high-frequency spectral flux or liveliness.

In addition, intersubject phase synchronization over time showed the strongest significant correlations (threshold of *Spearman’s* |*ρ*| > 0.300 and *p* < .05 / 17 features = .003) with the music set’s rhythmic and timbral features (Fig 3). Lower metrical centroid, which corresponds to slower pulsations or around 100 to 150 beats per minute (*ρ* = –0.454, *p* < .001), and higher metrical strength, which indicates clearer and stronger pulsations (*ρ* = 0.363, *p* < .001) were associated with greater movement synchrony. Moreover, spectral irregularity or variability (*ρ* = 0.410, *p* < .001), sensory dissonance or roughness (*ρ* = 0.358, *p* < .001), and high-frequency spectral flux or liveliness (*ρ* = 0.322, *p* < .001) were related to greater movement synchrony.

## Discussion

The present study examined influences on movement synchrony across a large number of individuals dancing in a real-world club environment. Movement acceleration was recorded from mobile devices placed on participants’ waists during a music set in a dance club, and different methods of measuring group synchrony of movement were evaluated to identify how it covaried with various features of the music set. During data acquisition for the assessment of the synchrony measures, *z*-aligned axis combination and intersubject phase synchronization provided the most sensitive and reliable measure of group movement synchrony. As the best compromise between discriminability and reliability, intersubject phase synchronization of movement data preprocessed using *z*-aligned axis combination, linear interpolation, averaging downsampling, and *coif1* wavelet decomposition–which displayed the highest agreement across two sets of devices on the same participants and ranked sixth on correlations with the synchrony and non-synchrony conditions–was used for analysis of data from the dance club event. During the club music set, group synchrony of torso movement was most strongly associated with pulsations that approximate walking rhythm (100–150 beats per minute) [36]. Songs with higher play counts were also correlated with greater group synchrony. Group synchrony thus appears to be constrained by familiarity of the movement (walking action and rhythm) and of the music (song popularity).

Shared intentionality arising from familiar music may promote group movement synchrony. Task-sharing, or knowing what others should do under certain stimulus conditions, enables better predictions of others’ actions and thus coordination of actions between individuals [37]. For example, children demonstrated greater drumming synchrony with a human partner compared to a drumming machine because the contingency and interactivity of a social condition [38] may be more likely to create a shared representation of the joint action task to play the drums together [39]. Similarly, rhythmic coordinated action such as in musical ensembles is facilitated by common performance goals based on knowledge about the musical structure and expressive intentions of other performers [40]. In an experiment with piano duos, body sway and keystroke coordination improved across repeat performances as more familiarity with a co-performer’s duet part and playing style was acquired [41]. The formation of an implicit common goal, and thus the anticipation of and coordination with others’ movements, is perhaps more likely to occur for popular songs as more people would have knowledge of how the songs progress or even a previously learned or oft-practiced motor sequence related to them. It is also possible that more people chose to dance, anticipating that others would join them, during the more popular songs compared to the less popular songs, during which some people may have chosen to stand on the sidelines and wait for another song they knew better. However, although shared intentionality arising from music familiarity appears to facilitate group movement synchrony, it remains unclear whether the resulting movement synchrony then leads to social bonding, movement synchrony simply reflects the resulting social bonding, or social bonding from shared intentionality leads to movement synchrony. This could be addressed in future studies by assessing changes in a variety of measures and over a wide range of epochs in both social bonding and group synchrony, which is usually only assumed under group assignments or instruction manipulations.

Additionally, the natural frequency of familiar movement such as walking constrains group movement synchrony. Smaller differences between the eigenfrequencies of two individuals’ movements facilitate greater interpersonal coordination [42]. For example, manipulating the natural frequency of pendulum swinging [43], chair rocking [44], sword swinging [45], and hand clapping [45] by placing different weights on the two performers resulted in decreased synchrony of movement. However, during side-by-side walking, interpersonal coordination was not influenced by differences in preferred stride frequency or variations in walking speed [46]. Thus, rhythms similar to walking (e.g., front-back movement, 100 to150 beats per minute) [36], which could be more common or effortless to perform while in a standing space, may be both easier to entrain to and easier to coordinate with other people. These results, however, might be specific to the waist placement of the accelerometer in the present study, since arm, leg, and head movements are likely to have different natural frequencies as would autonomic measures such as heart rate and breathing. It would be valuable for future studies to examine how variations in group synchrony of these other locations and measures are differentially influenced by music features as well has how they differentially affect the degree of social bonding.

One of the challenges of studying interpersonal synchrony is the lack of consensus on how to best quantify group coordination. Some experiments used group synchrony measures based on averaged pairwise statistics to compare across time or group conditions, including cross recurrence quantification [11], generalized partial directed coherence [13], phase synchronization index [12], and intersubject correlation [14–16]. These measures, however, did not allow the assessment of group synchrony at specific time points. Other studies computed variations in group synchrony over time, but the advantages of using averaged pairwise statistics (intersubject phase synchronization) [20,21] compared to averaged individual-relative-to-group indices (cluster phase method) [22,23] or only an overall group measure (Kuramoto order parameter) [47,48] have not been evaluated. Of the two continuous measures of group synchrony derived from individual data (intersubject phase synchronization and cluster phase method) examined in the present study, intersubject phase synchronization of overall movement weighted towards the front-back direction provided the most sensitive and reliable time courses of group synchrony. These results suggest that similarities among people’s movements might be more readily captured by correspondence between pairs of individuals than by the relationship of one individual to the rest of the group. Although intersubject phase synchronization has previously been used to quantify neural synchrony across participants as well as acceleration synchrony in the present study, it remains to be examined if the measure can also effectively represent group synchrony of other movement or autonomic time courses. It also remains to be investigated whether similarities among people’s movements are better reflected by correlation or coherence, which is based on variance information, or by phase difference, which is based on temporal information. A systematic comparison of the discriminability and reliability of different synchrony measures of various behavioral and physiological responses would further understanding of group synchrony.

In summary, movement synchrony across a large group of individuals in a real-world dance environment appears to be influenced by familiarity of the music (song popularity) and of the movement (walking action and rhythm). These high-level (e.g., shared intentionality of familiar music) and low-level (e.g., biomechanical constraints of familiar movement) influences on group synchrony revealed by the present findings suggest the importance of utilizing embodied and embedded approaches to studying large-scale social processes in the laboratory. These findings from a real-world, large-scale social and musical setting can guide the development of methods for capturing and examining collective experiences in the laboratory and for effectively linking them to synchrony across people in daily life.

## Supporting Information

S1 Fig Discriminability and reliability of group synchrony preprocessing and analysis parameters. *(top)* Discriminability was measured as the correlation of the group movement synchrony time course with the high and low synchrony conditions boxcar time course during the test session. *(bottom)* Reliability was measured as the absolute agreement between group movement synchrony time courses from two sets of devices on the same participants during the test session.
(TIF)

S2 Fig Group synchrony of movement for different song segments. Intersubject phase synchronization values were standardized for each song and then plotted across songs for each segment, including intro and outro, verse, pre-chorus and bridge, chorus, and interlude.
(TIF)

S1 File Song segments and group synchrony of movement during dance. (DOCX)

S1 Table Preprocessing and analysis pipelines with the highest discriminability. (DOCX)

S2 Table Preprocessing and analysis pipelines with the highest reliability. (DOCX)
