# Supplementary figures and images for: One in the Dance: Musical Correlates of Group Synchrony in a Real-World Club Environment
Source: PLoS One. 2016 Oct 20;11(10):e0164783. doi: 10.1371/journal.pone.0164783 (PMC5072606; doi:10.1371/journal.pone.0164783)

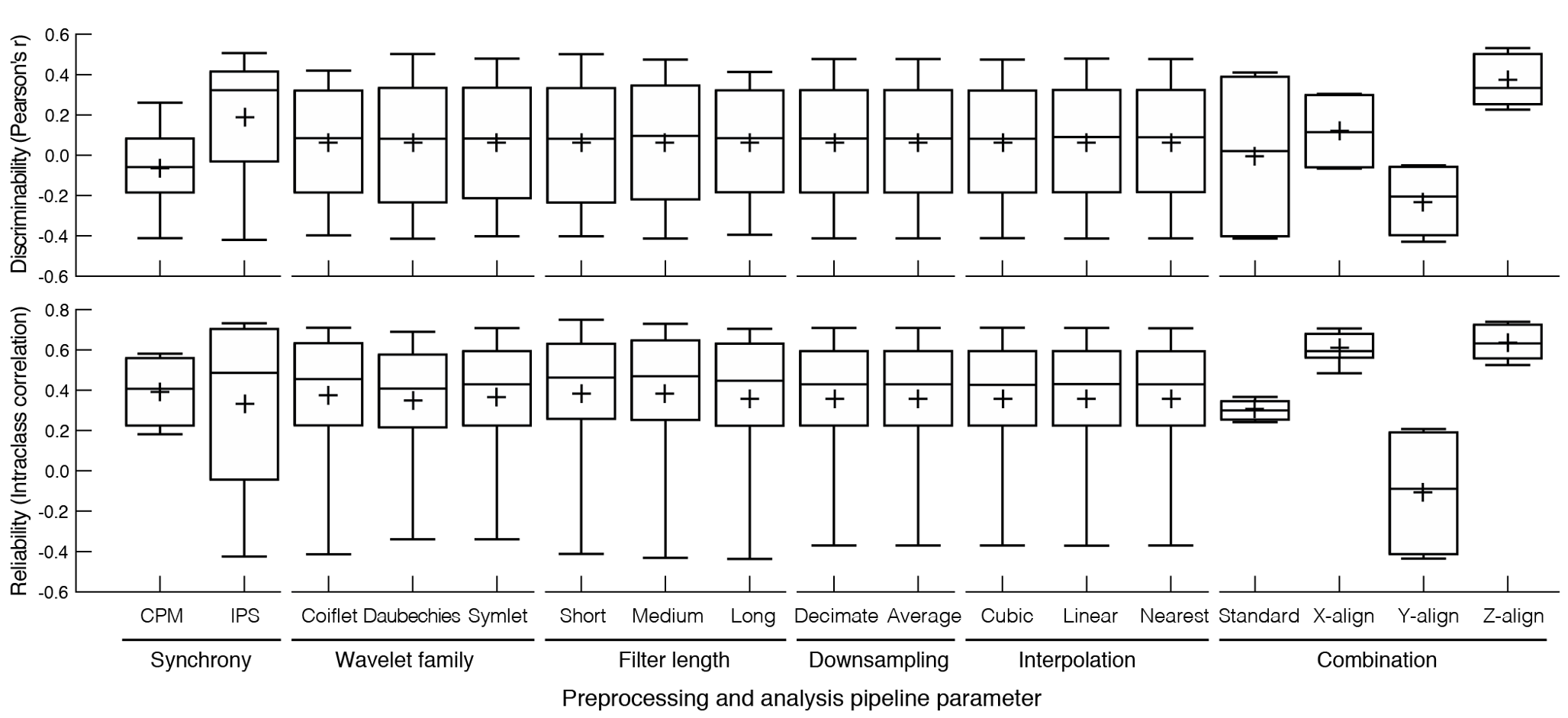

Supplement: S1 Fig — (top) Discriminability was measured as the correlation of the group movement synchrony time course with the high and low synchrony conditions boxcar time course during the test session. (bottom) Reliability was measured as the absolute agreement between group movement synchrony time courses from two sets of devices on the same participants during the test session. (TIF) [file pone.0164783.s001.tif]

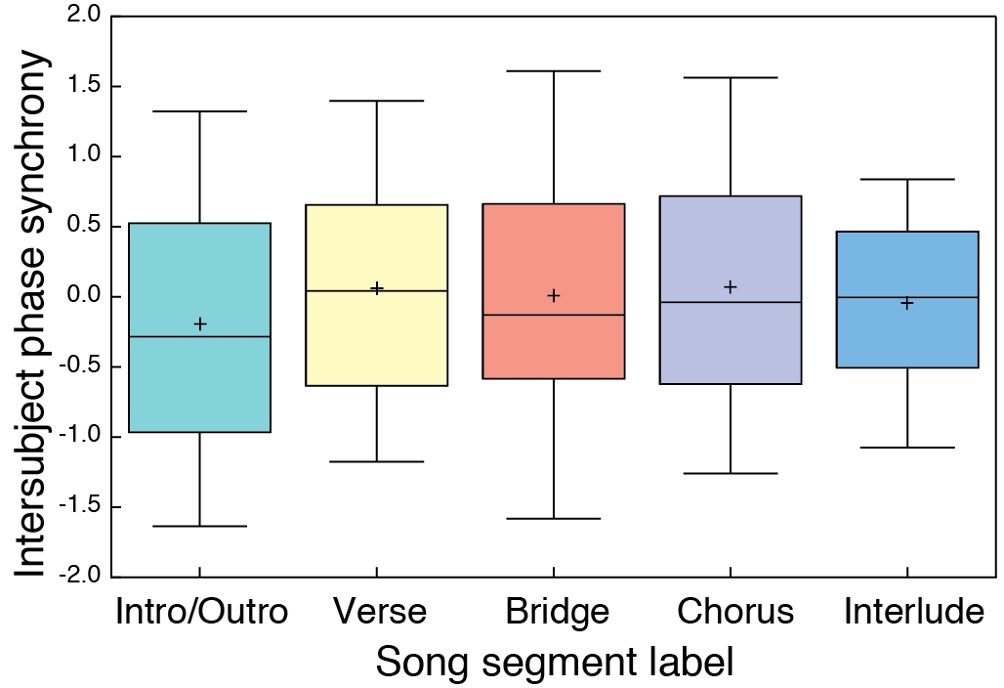

Supplement: S2 Fig — Intersubject phase synchronization values were standardized for each song and then plotted across songs for each segment, including intro and outro, verse, pre-chorus and bridge, chorus, and interlude. (TIF) [file pone.0164783.s002.tif]
