# Supplementary material for: One in the Dance: Musical Correlates of Group Synchrony in a Real-World Club Environment
Source: PLoS One. 2016 Oct 20;11(10):e0164783. doi: 10.1371/journal.pone.0164783 (PMC5072606; doi:10.1371/journal.pone.0164783)
Supplement: S1 File — (DOCX) [file pone.0164783.s003.docx]

**S1 File. Song segments and group synchrony of movement during dance.**

To assess the relationship between group movement synchrony and song segment type, time series regression corrected for autocorrelation (feasible generalized least squares for up to a lag of 5) [1–3] was performed on the intersubject phase synchronization time course with segments (intro/outro, verse, pre-chorus/bridge, chorus, and interlude) and songs (nine songs played during the music set) as covariates. None of the segment types significantly influenced group synchrony, *β* < 0.0004, *t*(96974) < 1.12, *p* > .05 (S2 Fig). In addition, a one-way repeated measures analysis of variance was conducted to compare the rate of change in group synchrony across song segments, as measured by the average slope per song of the least-squares regression line for the first half of each segment’s intersubject phase synchronization time course. The rate of change of group synchrony did not significantly differ between segment types, *F*(4,16) = 0.995, *p* > .05.

*References*

1. Davidson R, MacKinnon JG. Econometric Theory and Methods. New York: Oxford University Press; 2004.

2. Greene WH. Econometric Analysis. Essex: Pearson Education; 2012.

3. Hamilton JD. Time Series Analysis. Princeton: Princeton University Press; 1994.
