# Supplementary material for: One in the Dance: Musical Correlates of Group Synchrony in a Real-World Club Environment
Source: PLoS One. 2016 Oct 20;11(10):e0164783. doi: 10.1371/journal.pone.0164783 (PMC5072606; doi:10.1371/journal.pone.0164783)
Supplement: S1 Table — (DOCX) [file pone.0164783.s004.docx]

**S1 Table. Preprocessing and analysis pipelines with the highest discriminability.**

| *Parameters* | | | | | *Pearson’s r (95% CI)* |
| --- | --- | --- | --- | --- | --- |
| *Axes combination* | *Time interpolation* | *Data downsampling* | *Wavelet decomposition* | *Group synchrony measure* |  |
| zalign | linear | decimate | db6 | ips | 0.538 (0.535, 0.542) |
| zalign | linear | average | db6 | ips | 0.538 (0.535, 0.542) |
| zalign | nearest | decimate | db6 | ips | 0.538 (0.534, 0.542) |
| zalign | nearest | average | db6 | ips | 0.538 (0.534, 0.542) |
| zalign | linear | decimate | coif1 | ips | 0.534 (0.530, 0.537) |
| zalign | linear | average | coif1 | ips | 0.533 (0.530, 0.537) |
| zalign | nearest | decimate | coif1 | ips | 0.533 (0.529, 0.537) |
| zalign | nearest | average | coif1 | ips | 0.533 (0.529, 0.537) |
| zalign | cubic | average | db6 | ips | 0.532 (0.528, 0.536) |
| zalign | cubic | decimate | db6 | ips | 0.532 (0.528, 0.536) |
| zalign | cubic | decimate | coif1 | ips | 0.528 (0.524, 0.532) |
| zalign | linear | decimate | db4 | ips | 0.528 (0.524, 0.532) |
| zalign | cubic | average | coif1 | ips | 0.528 (0.524, 0.532) |
| zalign | linear | average | db4 | ips | 0.528 (0.524, 0.532) |
| zalign | nearest | decimate | db4 | ips | 0.527 (0.523, 0.531) |
| zalign | nearest | average | db4 | ips | 0.527 (0.523, 0.531) |
| zalign | cubic | average | db4 | ips | 0.521 (0.517, 0.525) |
| zalign | cubic | decimate | db4 | ips | 0.521 (0.517, 0.525) |
| zalign | linear | decimate | sym4 | ips | 0.506 (0.502, 0.510) |
| zalign | linear | average | sym4 | ips | 0.506 (0.502, 0.510) |
| *Note.* All correlations significant with *p* < .0001. | | | | | |
