# Supplementary material for: One in the Dance: Musical Correlates of Group Synchrony in a Real-World Club Environment
Source: PLoS One. 2016 Oct 20;11(10):e0164783. doi: 10.1371/journal.pone.0164783 (PMC5072606; doi:10.1371/journal.pone.0164783)
Supplement: S2 Table — (DOCX) [file pone.0164783.s005.docx]

**S2 Table. Preprocessing and analysis pipelines with the highest reliability.**

| *Parameters* | | | | | *Intraclass correlation (95% CI)* |
| --- | --- | --- | --- | --- | --- |
| *Axes combination* | *Time interpolation* | *Data downsampling* | *Wavelet decomposition* | *Group synchrony measure* |  |
| zalign | linear | average | coif1 | ips | 0.752 (0.746, 0.758) |
| zalign | linear | decimate | coif1 | ips | 0.752 (0.746, 0.758) |
| zalign | nearest | average | coif1 | ips | 0.751 (0.744, 0.757) |
| zalign | nearest | decimate | coif1 | ips | 0.751 (0.744, 0.757) |
| zalign | cubic | average | coif1 | ips | 0.749 (0.743, 0.755) |
| zalign | cubic | decimate | coif1 | ips | 0.749 (0.743, 0.755) |
| zalign | linear | average | sym4 | ips | 0.740 (0.734, 0.746) |
| zalign | linear | decimate | sym4 | ips | 0.740 (0.734, 0.746) |
| zalign | nearest | decimate | sym4 | ips | 0.739 (0.733, 0.746) |
| zalign | nearest | average | sym4 | ips | 0.739 (0.733, 0.746) |
| zalign | cubic | average | sym4 | ips | 0.737 (0.731, 0.743) |
| zalign | linear | average | db6 | ips | 0.737 (0.731, 0.742) |
| zalign | linear | decimate | db6 | ips | 0.737 (0.731, 0.742) |
| zalign | cubic | decimate | sym4 | ips | 0.737 (0.731, 0.742) |
| zalign | nearest | average | db6 | ips | 0.736 (0.730, 0.742) |
| zalign | nearest | decimate | db6 | ips | 0.736 (0.730, 0.742) |
| zalign | linear | decimate | coif3 | ips | 0.732 (0.725, 0.739) |
| zalign | cubic | average | db6 | ips | 0.732 (0.726, 0.737) |
| zalign | cubic | decimate | db6 | ips | 0.732 (0.726, 0.737) |
| zalign | linear | average | coif3 | ips | 0.732 (0.725, 0.739) |
| *Note.* All correlations significant with *p* < .0001. | | | | | |
